# Meta-analysis of the value of dual-energy computed tomography in the diagnosis of anterior cruciate ligament injuries of the knee

**DOI:** 10.1186/s12891-024-07632-6

**Published:** 2024-07-18

**Authors:** Qiao Lin, Jiwen Wu, Shijun Qiu

**Affiliations:** 1 First Clinical Medical College, Guangzhou University of Chinese Medicine, Guangzhou, 510405 People’s Republic of China; 2 Department of Radiology, The First Affiliated Hospital of Guangzhou University of Chinese Medicine, No.16, Airport Road, Baiyun District, Guangzhou, 510405 People’s Republic of China

**Keywords:** Dual-energy computed tomography, Diagnosis, Anterior cruciate ligament injuries, Meta-analysis

## Abstract

**Background:**

This meta-analysis assessed the efficacy of dual-energy computed tomography (DECT) in the diagnosis of anterior cruciate ligament (ACL) injuries.

**Methods:**

The literature search was performed up to December 8, 2023, and included a comprehensive examination of several databases: PubMed, Embase, Cochrane Library, Web of Science, China National Knowledge Infrastructure (CNKI), Wanfang, and VIP. Diagnostic metrics sensitivity, specificity, positive likelihood ratio (PLR), negative likelihood ratio (NLR), diagnostic odds ratio (DOR), and a summary receiver operating characteristic (SROC) were determined using a bivariate model analysis. Heterogeneity within the data was explored through subgroup analyses, which considered variables including geographical region, use of magnetic resonance imaging (MRI), arthroscopy, and study design.

**Results:**

The analysis included ten studies encompassing 544 patients. DECT demonstrated substantial diagnostic utility for ACL injuries of the knee, with a sensitivity of 0.91 (95% confidence interval [CI]: 0.88–0.94), a specificity of 0.90 (95% CI: 0.81–0.95), a PLR of 9.20 (95% CI: 4.50–19.00), a NLR of 0.10 (95% CI: 0.06–0.14), a DOR of 97.00 (95% CI: 35.00–268.00), and an area under the curve (AUC) of 0.95 (95% CI: 0.93–0.97). The subgroup analyses consistently showed high diagnostic precision for ACL injuries across Asian population (sensitivity: 0.91, specificity: 0.91, PLR: 9.90, NLR: 0.09, DOR: 105.00, AUC: 0.96), in MRI subgroup (sensitivity: 0.85, specificity: 0.94, PLR: 9.57, NLR: 0.18, DOR: 56.00, AUC: 0.93), in arthroscopy subgroup (sensitivity: 0.92, specificity: 0.89, PLR: 8.40, NLR: 0.09, DOR: 94.00, AUC: 0.95), for prospective studies (sensitivity: 0.92, specificity: 0.88, PLR: 7.40, NLR: 0.09, DOR: 78.00, AUC: 0.95), and for retrospective studies (sensitivity: 0.91, specificity: 0.93, AUC: 0.93).

**Conclusion:**

DECT exhibits a high value in diagnosing ACL injuries. The significant diagnostic value of DECT provides clinicians with a powerful tool that enhances the accuracy and efficiency of diagnosis and optimizes patient management and treatment outcomes.

## Background

The anterior cruciate ligament (ACL) plays a major role in knee proprioception and is responsible for maintaining knee joint stability and functionality [1, 2]. However, ACL injuries are one of the most common knee pathologies and are characterized by long convalescence periods and associated financial burdens [3]. The annual incidence of ACL injuries in the United States is 120,000 and continues to rise [4]. Quality of life is affected up to 5 years after an ACL injury [5]. Furthermore, ACL injuries are associated with an increased risk of post-traumatic knee osteoarthritis [6]. Therefore, accurate diagnosis is essential for the treatment and rehabilitation of patients with ACL injuries.

Arthroscopy is widely recognized as the gold standard for diagnosing ACL injuries [7, 8]. However, arthroscopy is not only expensive and slow but also traumatic to the patient [9, 10]. Magnetic resonance imaging (MRI) is recognized as a non-invasive diagnostic tool for the detection of ACL injuries [7, 11], however, have limitations in certain situations, particularly in the context of acute trauma and for participants with specific contraindications [7]. Spectral computed tomography (CT) represents a novel imaging approach that can noninvasively visualize, quantify, and characterize many musculoskeletal pathologies [12]. Dual-energy computed tomography (DECT), as a subset of spectral CT imaging, has revolutionized radiology by enabling material differentiation, superior tissue characterization, robust quantification, and a marked reduction in iodine dosage [13]. DECT overcomes many of the traditional limitations of CT and provides anatomical details previously only seen in MRI [14]. DECT can detect bone marrow edema [15], which is an important accompanying manifestation of ACL injuries. DECT has been explored for its potential role in diagnosing ligament injuries [16, 17]. In a study conducted at a level-one trauma center, the sensitivity and specificity of DECT in detecting ACL ruptures were found to be 79% and 100%, respectively [16]. A previous study by Gruenewald et al. indicated that DECT-derived color-coded collagen reconstructions significantly enhance diagnostic precision and certainty when evaluating the condition of the cruciate ligaments, as opposed to the conventional grayscale CT imaging used in acute trauma patients [17]. Another study has found that DECT demonstrates good diagnostic accuracy and reliability in the diagnosis of ACL injuries [18]. DECT, while offering improved image quality and material differentiation, still involves ionizing radiation [19]. Thereby, there is a need for evidence regarding the value of DECT in the diagnosis of ACL injuries. However, there has been no published meta-analysis assessing the application of DECT in ACL injury diagnostics. A meta-analytic synthesis of existing research findings is warranted to address this gap.

Herein, the meta-analysis evaluates the value of DECT in diagnosing ACL injuries. Accurate diagnosis may be crucial for the management of ACL injuries.

## Methods

This study was conducted in accordance with the PRISMA (Preferred Reporting Items for Systematic Reviews and Meta-Analyses) criteria [20].

### Literature search

The search was conducted up to December 8, 2023, and encompassed the following databases: PubMed, Embase, Cochrane Library, Web of Science, China National Knowledge Infrastructure (CNKI), Wanfang, and VIP. The PubMed search strategy is as follows: “Radiography, Dual-Energy Scanned Projection” OR “Digital Scanned Projection Radiography, Dual Energy” OR “Dual-Energy Scanned Projection Radiography” OR “Digital Scan Projection Radiography, Dual-Energy” OR “Dual Energy Scanned Projection Radiography” OR “Radiography, Dual Energy Scanned Projection” OR “Digital Scan Projection Radiography, Dual Energy” OR “Digital Scanned Projection Radiography, Dual-Energy” OR “Dual-energy” OR “Dual energy” OR “DECT” OR “Tomography, x-ray computed” OR “X-Ray Computed Tomography” OR “Tomography, X-Ray Computerized” OR “Tomography, X Ray Computerized” OR “Computed X Ray Tomography” OR “X-Ray Computer Assisted Tomography” OR “X Ray Computer Assisted Tomography” OR “Tomography, X-Ray Computer Assisted” OR “Tomography, X Ray Computer Assisted” OR “Computerized Tomography, X Ray” OR “Computerized Tomography, X-Ray” OR “X-Ray Computerized Tomography” OR “CT X Ray*” OR “Tomodensitometry” OR “Tomograph*, X Ray Computed” OR “CAT Scan*, X Ray” OR “Tomography, Transmission Computed” OR “Computed Tomography, Transmission” OR “Transmission Computed Tomography” OR “CT Scan*, X-Ray” OR “Computed Tomography, X-Ray” OR “Computed Tomography, X Ray” OR “X Ray Computerized Tomography” OR “Cine-CT” OR “Cine CT” OR “Electron Beam Computed Tomography” OR “Electron Beam Tomography” OR “Beam Tomography, Electron” OR “Tomography, Electron Beam” OR “Tomography, X-Ray Computerized Axial” OR “Tomography, X Ray Computerized Axial” OR “X-Ray Computerized Axial Tomography” OR “X Ray Computerized Axial Tomography” OR “computer tomography” OR “CT” AND “Anterior Cruciate Ligament*”. The identified literature was imported into EndNote X9, where an initial screening was performed by reviewing the titles and abstracts. Following this preliminary culling, the remaining articles were subjected to a full-text review to exclude those that did not meet the inclusion criteria. The final selection of articles that fulfilled the study’s requirements was then incorporated into the meta-analysis.

### Inclusion and exclusion criteria

The inclusion criteria were based on the PICOS (Patient, Intervention, Comparison, Outcome, and Study Design) framework: (1) patient: patients suspected of having an ACL injury of the knee; (2) intervention and comparison: patients underwent DECT examination; (3) outcome: the outcomes of sensitivity, specificity, positive likelihood ratio (PLR), negative likelihood ratio (NLR), diagnostic odds ratio (DOR), and a summary receiver operating characteristic (SROC); (4) study design: cohort study; (5) Chinese and English literature.

Exclusion criteria: (1) animal experiments; (2) research unrelated to the topic; (3) case reports, editorial materials, conference abstracts, protocols, guidelines, expert consensus documents, reviews, and meta-analyses.

### Data collection

Two reviewers (Qiao Lin, and Jiwen Wu) independently collected data from the selected studies. The data extracted from the eligible studies included details such as the author's name, publication year, country where the study was conducted, study design, specific inclusion criteria used, sample size, participant age in years, gender distribution (male and female), the number of DECT readers involved, the Kappa statistic for inter-rater reliability, years of experience of the DECT readers, and the CT protocol utilized in the studies. In instances of discrepancy, consensus was reached by referring to a third investigator (Shijun Qiu) for arbitration.

### Quality assessment

The quality of the literature was assessed using the Quality Assessment of Diagnostic Accuracy Studies-2 (QUADAS-2) [21] tool, which is a standardized instrument for evaluating the quality of diagnostic accuracy studies. The assessment was conducted by reflecting on the risk of bias and the applicability concerns. The risk of bias included domains such as patient selection, index test, reference standard, flow and timing, with each domain being rated as “high”, “low”, or "unclear." The applicability concerns were judged based on the domains of patient selection, index test, and reference standard, with each being assessed for the degree of alignment with the review question using the same “high”, “low”, or “unclear” criteria.

### Statistical analysis

The data analysis was conducted using Meta-Disc 1.4, Stata 15.1, and RevMan 5.4 software. Results were obtained through direct extraction or indirect calculation. The presence of threshold effects in the studies was assessed using Meta-Disc 1.4 software. If there is a strong positive correlation between the logit of sensitivity and logit of 1-specificity (*P* < 0.05), assessed by Spearman’s correlation coefficients, threshold effects were present. Stata 15.1 software was utilized for bivariate model analysis to evaluate outcomes such as sensitivity, specificity, PLR, NLR, and DOR, and to generate the SROC curve. RevMan 5.4 software was employed to create graphical representations for the quality assessment of the literature. Subgroup analysis was used to explore heterogeneity based on the region, MRI, arthroscopy, and study design.

## Results

### Process of study selection and characteristics of included studies

**Fig. 1:**
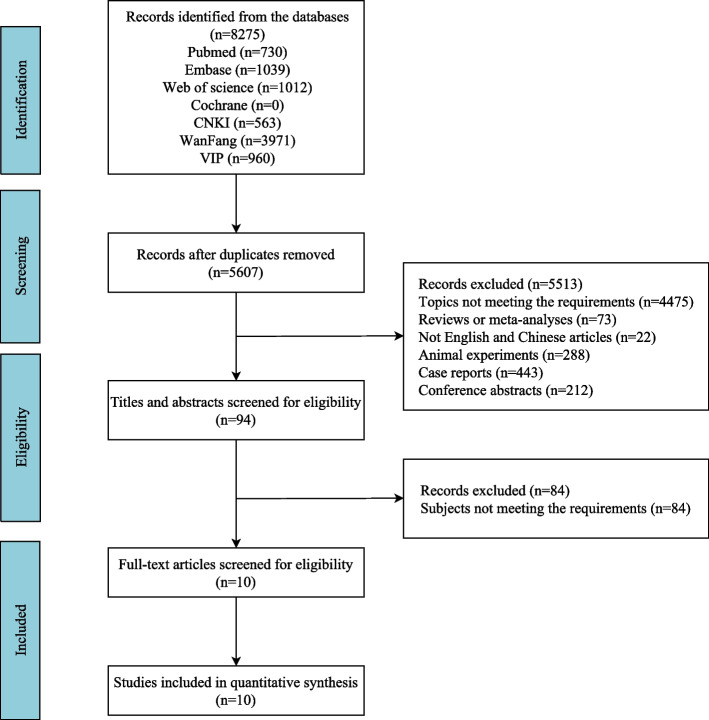
The flowchart illustrates the process of study selection

**Fig. 2:**
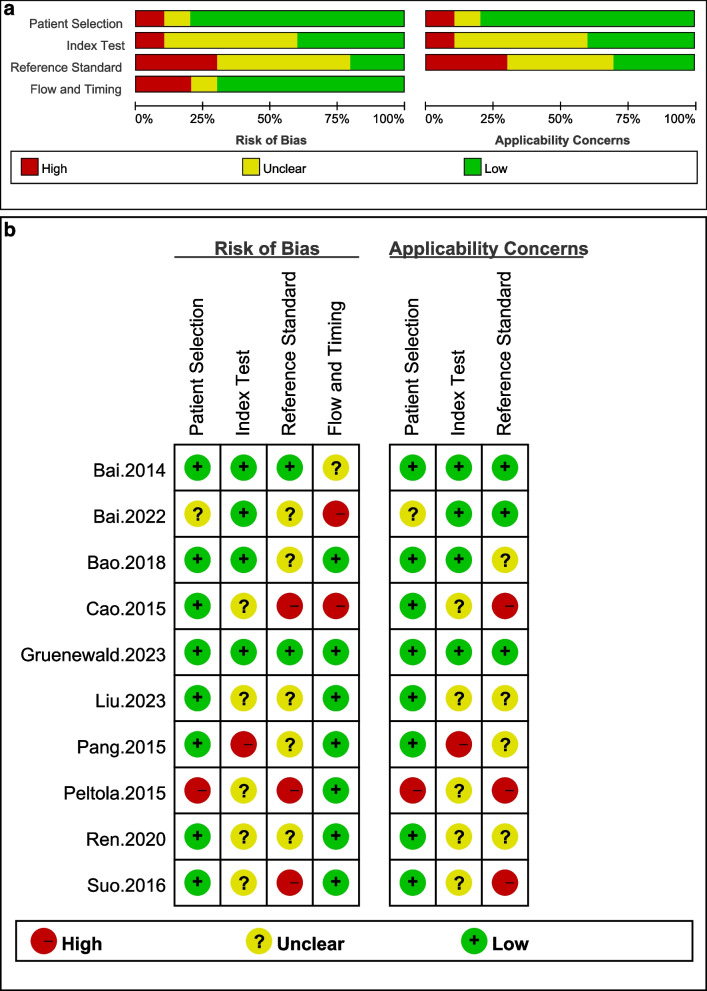
Quality assessment graph; 2a, the methodological quality graph; 2b, assessment of the risk of bias and applicability concerns for each study

The initial search across various databases yielded a total of 8,275 records. These were sourced from PubMed (730), Embase (1,039), Web of Science (1,012), Cochrane (0), CNKI (563), WanFang (3,971), and VIP (960). After removing duplicate records, the number of records was reduced to 5,607. From the remaining records, 94 were screened for eligibility based on their titles and abstracts. Out of the screened records, 10 full-text articles were assessed for eligibility. Ultimately, 10 studies [17, 18, 22–29] were included in the quantitative synthesis. Figure 1 depicts the study selection process. The included studies, which span from 2014 to 2023, involved a variety of countries, including Germany, China, and Finland. The studies were categorized into two main types: four were retrospective studies, and six were prospective studies. By aggregating the participant counts across all ten studies, the total number of patients assessed amounts to 544. Table 1 summarizes the characteristics of the included studies. Figure 2a shows the methodological quality graph. Figure 2b provides an individual assessment of the risk of bias and applicability concerns for each study.

**Table 1:** Characteristics of included studies

Author	Year	Country	Study design	Inclusion criteria	No. patients	Age (year)	Sex (male/female)	No. readers	Kappa	Reader experience (years)	CT protocol
Gruenewald	2023	Germany	Retrospective study	Adults 18 + with acute knee trauma (within 3 days)	85	44.5 ± 16.0	50/35	5	NA	Consecutive CTs read by 5 radiologists: 3 certified (C.B., I.Y., S.M.), 2 in training (L.G., V.K.), 1–7 years in musculoskeletal imaging	Third-gen dual-source CT (Somatom Force) in dual-energy mode: 90 kVp/180 mAs (Tube A), Sn150 kVp/180 mAs (Tube B, 0.64-mm tin filter), no contrast
Liu	2023	China	Prospective study	Ages ≥ 14, non-pregnant, unilateral ACL injury, positive physical exam; eligible with concomitant meniscal/chondral lesions	51	27 ± 8.7	31/20	2	NA	Reader 1: with 14 years of experience in orthopedics and sports medicine; Reader 2: with 11 years of experience in musculoskeletal radiology	Somatom Drive DECT scans, dual-energy mode, lead shielding; simultaneous bilateral knee scans with dual kV settings (Tube A: 80 kV, 210 mAs; Tube B: Sn140 kV, 105 mAs); Protocol: 1.0 mm slice, 0.5 s rot. time, 32 × 0.6 mm collimation, 0.7 pitch
Bai	2022	China	Retrospective study	Patients with knee injury	35	29.3 ± 9.5	27/8	2	Inter group: 0.836	Imaging physician with at least 10 years of experience and a professional title of Deputy Chief Physician	Siemens dual-source spiral CT, dual-energy, low-dose: Tube A 140 kV/123mAs, Tube B 80 kV/29mAs, 20 × 0.6 mm collimation, 0.7 pitch, 250 mm FOV. Reconstruction: 1 mm layer, 0.7 mm increment, D30s algorithm, limb preset window
Ren	2020	China	Prospective study	Unilateral knee discomfort with symptoms (swelling, pain, instability, limited ROM); positive findings in front drawer, Lachman, and pivot shift tests	36	33.5 ± 10.8	21/15	1	Inter group: 0.719	Deputy chief physician	Siemens 64-row CT, dual-energy mode. Supine position, knees extended and together. Scan range: femoral condylar margin to fibular capitulum
Bao	2018	China	Prospective study	Patients with cruciate ligament injury of knee joint	70	30.54 ± 5.26	52/18	NA	NA	NA	German Siemens 64-row dual-source CT with post-processing workstation: supine position, knees extended and closed. Scan range: bilateral knee joints to femoral condyle upper edge, tibial condyles, and fibular head lower edge. Dual-energy volume scan, params: Tube A 140 kV/234 mA, Tube B 80 kV/56 mA; Pitch 0.7 mm; Layer thickness 0.75 mm; Recon increment 0.5 mm; FOV 250 mm
Suo	2016	China	Prospective study	Patients with knee injuries caused by different causes	30	38 (14–65)^a^	20/10	3	Inter group: 0.8	Deputy Chief Physician or higher title	Scanning params: Tube A 140 kV/45 mA, Tube B 80 kV/191 mA, collimation 40 × 0.6 mm, pitch 0.7, recon layer thickness 0.6 mm, FOV 256 mm. Scan range: from femoral superior condyle to tibial condyle and fibular capella lower margin
Cao	2015	China	Prospective study	Patients with acute knee joint trauma	30	35.2 ± 9.3	22/8	2	Within group: 0.756	Two doctors with more than 10 years of experience in CT and MR work	CT: Siemens Dual Source CT (Somatom Definition), dual-energy mode. Tubes: A 140 kV/60mAs, B 80 kV/25mAs. Frame speed: 0.5 s/rpm. Pitch: 0.9. Collimation: 20 × 0.6 mm. CTDIvol: 11.88 mGy
Pang	2015	China	Prospective study	Patients with suspected anterior cruciate ligament injury of the knee	100	15–55^a^	59/41	4	Inter group: 0.624	Reviewed by 2 senior orthopedic surgeons, 2 radiologists	Siemens dual-source dual-energy CT scanned both knee joints. Params: 140 kV and 80 kV tube voltages, 4D auto current regulation, 20 mm × 0.6 mm collimation, 0.6 mm pitch, 250 mm FOV
Peltola	2015	Finland	Retrospective study	Acute or subacute (< 10 days) knee trauma, with DECT and MRI during the acute/subacute phase	18	36 (18–63)^a^	8/10	2	Within group: 0.85; Inter group: 0.80	Dual readings by two radiologist authors: one with 2 years, the other with over 10 years in musculoskeletal radiology	GE Discovery CT 750 HD DECT images: 0.516 pitch, 0.5-s rotation, 32 × 0.625-mm collimation, 512 × 512 matrix. Tube voltage 80–140 kV, max 630 mA
Bai	2014	China	Retrospective study	Patients with cruciate ligament injury of knee joint	89	26.7 ± 11.07	74/15	2	NA	Image analysis by an experienced radiologist (Associate Chief or higher) and an osteopathic arthrologist on patients' knee joint dual-energy CT data,	Scanning parameters: Tube currents 30mAs and 128mAs, tube voltages 140 kV and 80 kV, pitch 0.7, collimation 0.6 mm, recon layer thickness 0.75 mm, FOV 250 mm, CTDI 5.96 mGy. Scan range: femoral superior condyle to tibial condyle and fibular capitis lower margin

*ACL* Anterior cruciate ligament, *CT* Computed tomography, *MRI* Magnetic resonance imaging, *DECT* Dual-energy computed tomography, *FOV* Field of View, *GE* General Electric, *NA* Not applicable

^a^Media (range)

### Meta-analysis of DECT in diagnosing ACL injuries

#### Overall

The meta-analysis revealed no threshold effect with a Spearman correlation coefficient of *r* = -0.122 and *P* = 0.738. The bivariate model yielded a pooled sensitivity of 0.91 [95% confidence interval (CI): 0.88–0.94], specificity of 0.90 (95% CI: 0.81–0.95), PLR of 9.20 (95% CI: 4.50–19.00), NLR of 0.10 (95% CI: 0.06–0.14), DOR of 97.00 (95% CI: 35.00–268.00), and area under the curve (AUC) of 0.95 (95% CI: 0.93–0.97), indicating that DECT held significant diagnostic value for ACL injuries of the knee (Table 2).

**Table 2:** Meta-analysis of DECT in diagnosing ACL injuries

Outcomes	Sensitivity	Specificity	PLR	NLR	DOR	AUC	Threshold effect
Overall	0.91 (0.88, 0.94)	0.90 (0.81, 0.95)	9.20 (4.50, 19.00)	0.10 (0.06, 0.14)	97.00 (35.00, 268.00)	0.95 (0.93, 0.97)	*r* = -0.122, *P* = 0.738
Asia	0.91 (0.87, 0.94)	0.91 (0.81, 0.96)	9.90 (4.40, 22.00)	0.09 (0.06, 0.15)	105.00 (33.00, 330.00)	0.96 (0.93, 0.97)	*r* = -0.371, *P* = 0.365
MRI	0.85 (0.71, 0.94)	0.94 (0.79, 0.99)	9.57 (2.93, 31.28)	0.18 (0.09, 0.36)	56.00 (12.00, 260.00)	0.93 (0.82, 1.00)	*r* = 0.500, *P* = 0.667
Arthroscopy	0.92 (0.88, 0.95)	0.89 (0.77, 0.95)	8.40 (3.70, 19.20)	0.09 (0.05, 0.15)	94.00 (28.00, 319.00)	0.95 (0.93, 0.97)	*r* = -0.500, *P* = 0.253
Prospective study	0.92 (0.86, 0.95)	0.88 (0.70, 0.96)	7.40 (2.70, 20.50)	0.09 (0.05, 0.18)	78.00 (16.00, 373.00)	0.95 (0.93, 0.97)	*r* = -0.300, *P* = 0.624
Retrospective study	0.91 (0.84, 0.94)	0.93 (0.74, 0.98)	12.50 (3.10, 50.60)	0.10 (0.06, 0.18)	122.00 (23.00, 653.00)	0.93 (0.90, 0.95)	*r* = -0.200, *P* = 0.800

*MRI* Magnetic resonance imaging, *DECT* Dual-energy computed tomography, *AUC* Area under curve, *DOR* Diagnostic odds ratio, *NLR* Negative likelihood ratio, *PLR* Positive likelihood ratio

#### Subgroup analyses of DECT in diagnosing ACL injuries

In the Asian subgroup of the analysis, the threshold effect was absent, as indicated by a Spearman correlation coefficient of *r* = -0.371 with *P* = 0.365. The bivariate model demonstrated a combined sensitivity of 0.91 (95% CI: 0.87–0.94) and specificity of 0.91 (95% CI: 0.81–0.96), with a PLR of 9.90 (95% CI: 4.40- 22.0), an NLR ratio of 0.09 (95% CI: 0.06–0.15), and a DOR of 105 (95% CI: 33.00–330.00). The AUC was 0.96 (95% CI: 0.93–0.97), confirming the high diagnostic utility of DECT for diagnosing ACL injuries in the knee among the Asian population (Table 2).

For the MRI subgroup, the analysis using Meta-Disc 1.4 software indicated no threshold effect with a Spearman correlation coefficient of *r* = 0.500 and *P* = 0.667. The bivariate model results showed a pooled sensitivity of 0.85 (95% CI: 0.71–0.94) and specificity of 0.94 (95% CI: 0.79–0.99), a PLR of 9.57 (95% CI: 2.93–31.28), and an NLR of 0.18 (95% CI: 0.09–0.36), a DOR of 56.00 (95% CI: 12.00–260.00), and AUC of 0.93 (95% CI: 0.82–1.00) (Table 2).

In the arthroscopy subgroup, the absence of threshold effect was also observed with a Spearman correlation coefficient of *r* = -0.500 and *P* = 0.253. The pooled sensitivity and specificity were 0.92 (95% CI: 0.88–0.95) and 0.89 (95% CI: 0.77–0.95), respectively, with a PLR of 8.40 (95% CI: 3.70–19.20) and a NLR of 0.09 (95% CI: 0.05–0.15), resulting in a DOR of 94.00 (95% CI: 28.00–319.00). The AUC was 0.95 (95% CI: 0.93–0.97), indicating a high diagnostic value of DECT for ACL injuries of the knee (Table 2).

For prospective studies, no threshold effect was detected with a Spearman correlation coefficient of *r* = -0.300 and *P* = 0.624. The pooled sensitivity and specificity were 0.92 (95% CI: 0.86–0.95) and 0.88 (95% CI: 0.70–0.96), respectively, with a PLR of 7.40 (95% CI: 2.70–20.50) and a NLR of 0.09 (95% CI: 0.05–0.18), leading to a DOR of 78.00 (95% CI: 16.00–373.00). The AUC was 0.95 (95% CI: 0.93–0.97), reinforcing the high diagnostic accuracy of DECT in diagnosing ACL injuries (Table 2).

For retrospective studies, the threshold effect was not present, as evidenced by a Spearman correlation coefficient of *r* = -0.200 and *P* = 0.800. The pooled sensitivity and specificity were 0.91 (95% CI: 0.84–0.94) and 0.93 (95% CI: 0.74–0.98), respectively. The positive and negative likelihood ratios were 12.50 (95% CI: 3.10–50.60) and 0.10 (95% CI: 0.06–0.18), respectively, with a DOR of 122.00 (95% CI: 23.00–653.00). The AUC was 0.93 (95% CI: 0.90–0.95), further supporting the high diagnostic value of DECT in the diagnosis of ACL injuries (Table 2).

## Discussion

This meta-analysis included ten eligible studies to assess the value of DECT in diagnosing ACL injuries. Overall, the analysis provided a pooled sensitivity of 0.91 and specificity of 0.90, with a PLR of 9.20, NLR of 0.10, DOR of 97.00, and AUC of 0.95, highlighting the significant diagnostic value of DECT for knee ACL injuries. Across different subgroup analyses, DECT has shown a high level of diagnostic accuracy for ACL injuries, with consistent results indicating its potential as a valuable diagnostic tool in the knee joint injury assessment.

DECT involves acquiring CT attenuation data at two distinct energy levels [30], and offers significant advantages over conventional CT in the musculoskeletal environment by providing additional information regarding tissue composition, reduced artifacts, and image optimization [31]. DECT is gaining increasing popularity and value in the field of musculoskeletal imaging [32]. Previous studies have confirmed the diagnostic value of DECT for knee joint injuries and ligaments. A study exploring the clinical application of DECT in the knee joint ligaments posits that DECT is a novel and valuable tool for the qualitative depiction of the major ligaments in the knee [33]. A retrospective, monocentric study revealed that DECT is readily accessible and can serve as a screening tool for the detection or exclusion of cruciate ligament injuries in patients with acute trauma [34]. In the study involving patients with acute trauma who underwent third-generation dual-source DECT, the author revealed that DECT-based colored collagen maps provided superior visualization of ligament integrity, enabling better detection of partial and complete tears [17]. A case–control study has found that DECT knee images reconstructed in the oblique sagittal plane using mixed kV or bone subtraction display (DECT or single-energy (SE)) can delineate subacute or chronic ruptures of the ACL [35]. To our knowledge, our meta-analysis represents the first systematic evaluation of the diagnostic value of DECT in the diagnosis of ACL injuries.

Our findings may provide a solid foundation for further investigation into the refinement of DECT techniques, the expansion of its applications, and the potential development of novel imaging biomarkers.

While MRI is widely recognized as the diagnostic modality of choice for a multitude of musculoskeletal disorders, accessibility to this method is not universally attainable for all patient populations [12]. Additionally, MRI is characterized by its protracted procedural duration, economic exigency, and constrained applicability within the spectrum of medical institutions. An ex-vivo experiment demonstrated that both DECT and MRI are equivalent in the depiction of the ACL and DECT may serve as an alternative to MRI for certain indications in the diagnosis of knee ligament injuries [36]. Another study supported that the DECT could effectively and reliably diagnose ACL ruptures using both qualitative and quantitative methods, potentially emerging as a promising alternative to MRI [18]. Our subgroup analysis further demonstrates that DECT is not inferior to MRI or arthroscopy in diagnosing ACL injuries. The findings of our study suggest that DECT may serve as a potential alternative to MRI or arthroscopy, garnering future attention due to its broader accessibility, reduced cost, and shorter scanning time.

In this meta-analysis, DECT has demonstrated high sensitivity and specificity in diagnosing ACL injuries. The sensitivity of a test, also known as the true positive rate, refers to its capacity to accurately detect and confirm the presence of a disease in individuals who have it [37]. The specificity of a test, alternatively termed the true negative rate, quantifies the test’s precision in correctly identifying individuals who are free from the disease [37]. DECT exhibits high sensitivity and specificity in diagnosing ACL injuries, signifying that DECT can accurately detect ACL injuries that are truly present (reducing false negatives) and correctly rule out ACL injuries when they are not present (reducing false positives). Such a diagnostic test is of significant value to clinicians as it offers dependable and precise diagnostic information, which is instrumental in guiding treatment decisions. High sensitivity ensures that DECT is likely to capture all cases of ACL injuries, minimizing the chance of missing out on crucial diagnoses. This is particularly important in the early stages of injury assessment when timely intervention can prevent further damage and facilitate better outcomes. On the other hand, high specificity means that DECT is less likely to indicate the presence of an ACL injury when none exists, thus avoiding unnecessary treatments and the associated risks and costs. This is crucial for managing patient expectations and ensuring that resources are allocated appropriately. The combination of high sensitivity and specificity in DECT makes it a robust diagnostic tool for ACL injuries, providing clinicians with the confidence to make informed decisions regarding patient care and treatment strategies.

The high diagnostic performance of DECT has a profound impact on clinical decision-making, particularly in the diagnosis and management of ACL injuries. Firstly, DECT may be an ideal initial screening tool for ACL injuries. By providing detailed images of soft tissues, including ligaments, DECT can quickly identify the presence of an injury, which is crucial for early intervention. As an initial screening tool, DECT may lead to more timely treatment plans, potentially reducing the risk of further damage and improving patient outcomes. Secondly, in situations where MRI is not available or contraindicated due to various reasons, DECT can serve as a reliable alternative. Its non-invasive nature and relatively lower cost compared to MRI make it a more accessible option for patients and healthcare systems. Thirdly, DECT’s ability to provide high-quality images may reduce the need for additional diagnostic procedures, such as arthroscopy, which is invasive and carries its own set of risks and costs. Fourthly, in regions where access to advanced imaging technologies like MRI is limited, DECT may bridge the gap. Its portability and the widespread availability of CT scanners make DECT a more feasible option for diagnosing ACL injuries. This can lead to better management of sports injuries in areas that previously lacked the necessary diagnostic tools. Fifthly, the cost of MRI can be a significant barrier in healthcare systems with limited resources. DECT offers a more cost-effective alternative without compromising on diagnostic accuracy. By reducing the financial burden on patients and healthcare facilities, DECT can make high-quality ACL injury assessments more widely available. Sixthly, our meta-analysis not only facilitates the adoption of DECT technology in clinical practice but also lays the groundwork for future research directions and further clinical trials. Through such systematic reviews and analyses, healthcare professionals can gain a better understanding of the accuracy and reliability of DECT in diagnosing ACL injuries, thereby providing patients with higher-quality medical services.

However, this meta-analysis still has several limitations. Firstly, the inclusion of only Chinese and English literature may introduce a language bias, potentially excluding relevant studies published in other languages that could have provided a more comprehensive understanding of DECT’s diagnostic performance in ACL injuries. Secondly, some of the results showed high levels of heterogeneity. Due to limitations in the literature, it was not possible to fully explore all sources of this heterogeneity. The variability could be attributed to differences in the number and experience of radiologists interpreting the scans in the original studies, as well as differences in age and gender distribution among the study populations. Thirdly, there was a scarcity of research on certain outcomes, which may affect the stability of the results. A larger body of evidence is needed to confirm the findings and to provide a more robust assessment of DECT’s role in diagnosing ACL injuries. Fourthly, due to constraints in the original literature, further exploration of the diagnostic efficacy of DECT in identifying specific types of ACL injuries was not possible. This limitation may impact the generalizability of DECT in clinical settings, as understanding its performance across different injury types is crucial for its broader adoption and application in the diagnosis and management of ACL injuries.

## Conclusion

Our findings suggest that the application of DECT imaging processing can serve as an important radiologic ancillary examination for the diagnosis of ACL injuries. Utilizing DECT CT imaging measurements allows for the initial diagnosis and precise localization of ACL injuries. DECT holds promise as an alternative to MRI or arthroscopy, offering a potential substitute in contexts where these traditional methods are either unavailable or impractical.

## Data Availability

The datasets used and/or analyzed during the current study are available from the corresponding author on reasonable request.

## Abbreviations

ACL: Anterior cruciate ligament
MRI: Magnetic resonance imaging
DECT: Dual-energy computed tomography
CNKI: China National Knowledge Infrastructure
PLR: Positive likelihood ratio
NLR: Negative likelihood ratio
DOR: Diagnostic odds ratio
SROC: Summary receiver operating characteristic
QUADAS-2: Quality Assessment of Diagnostic Accuracy Studies-2
CI: Confidence interval
AUC: Area under the curve

## References

[1] Fleming JD, Ritzmann R, Centner C (2022). Effect of an anterior cruciate ligament rupture on knee proprioception within 2 years after conservative and operative treatment: a systematic review with meta-analysis. Sports Med.

[2] Shom P, Varma AR, Prasad R (2023). The anterior cruciate ligament: principles of treatment. Cureus.

[3] Maniar N, Cole MH, Bryant AL, Opar DA (2022). Muscle force contributions to anterior cruciate ligament loading. Sports Med.

[4] Kaeding CC, Léger-St-Jean B, Magnussen RA (2017). Epidemiology and diagnosis of anterior cruciate ligament injuries. Clin Sports Med.

[5] Kvist J, Pettersson M (2024). Knee-related quality of life compared between 20 and 35 years after an anterior cruciate ligament injury treated surgically with primary repair or reconstruction, or nonsurgically. Am J Sports Med.

[6] Evers BJ, Van Den Bosch MHJ, Blom AB, van der Kraan PM, Koëter S, Thurlings RM (2022). Post-traumatic knee osteoarthritis; the role of inflammation and hemarthrosis on disease progression. Front Med (Lausanne).

[7] Zhao M, Zhou Y, Chang J, Hu J, Liu H, Wang S (2020). The accuracy of MRI in the diagnosis of anterior cruciate ligament injury. Ann Transl Med.

[8] Ji C, Chen Y, Zhu L, Zhang J (2021). Arthroscopic anterior cruciate ligament injury in clinical treatment of joint complications and CT observation. J Healthc Eng.

[9] Siemieniuk RAC, Harris IA, Agoritsas T, Poolman RW, Brignardello-Petersen R, Van de Velde S (2018). Arthroscopic surgery for degenerative knee arthritis and meniscal tears: a clinical practice guideline. Br J Sports Med.

[10] Nin DZ, Chen Y-W, Talmo CT, Hollenbeck BL, Niu R, Chang DC (2023). Arthroscopic procedures are performed in 5% of patients with knee osteoarthritis 1 year preceding total knee arthroplasty and are associated with increased stiffness and increased costs. Arthroscopy, Sports Medicine, and Rehabilitation.

[11] Sultana N, Shirin M, Jabeen S, Faruque MA, Sarkar SK, Nag UK (2023). Diagnostic accuracy of magnetic resonance imaging in evaluation of anterior cruciate ligament tear. Mymensingh Med J.

[12] Eibschutz LS, Matcuk G, Chiu MK-J, Lu MY, Gholamrezanezhad A (2024). Updates on the applications of spectral computed tomography for musculoskeletal imaging. Diagnostics.

[13] So A, Nicolaou S (2021). Spectral computed tomography: fundamental principles and recent developments. Korean J Radiol.

[14] Meer E, Patel M, Chan D, Sheikh AM, Nicolaou S (2023). Dual-energy computed tomography and beyond: musculoskeletal system. Radiol Clin North Am.

[15] Simonetti I, Verde F, Palumbo L, Di Pietto F, Puglia M, Scaglione M (2021). Dual energy computed tomography evaluation of skeletal traumas. Eur J Radiol.

[16] Peltola EK, Koskinen SK (2015). Dual-energy computed tomography of cruciate ligament injuries in acute knee trauma. Skeletal Radiol.

[17] Gruenewald LD, Koch V, Martin SS, Yel I, Mahmoudi S, Bernatz S (2023). Diagnostic value of DECT-based colored collagen maps for the assessment of cruciate ligaments in patients with acute trauma. Eur Radiol.

[18] Liu D, Hu P, Cai ZJ, Lu WH, Pan LY, Liu X (2023). Valid and reliable diagnostic performance of dual-energy CT in anterior cruciate ligament rupture. Eur Radiol.

[19] Henzler T, Fink C, Schoenberg SO, Schoepf UJ (2012). Dual-energy CT: radiation dose aspects. AJR Am J Roentgenol.

[20] Page MJ, McKenzie JE, Bossuyt PM, Boutron I, Hoffmann TC, Mulrow CD (2021). The PRISMA 2020 statement: an updated guideline for reporting systematic reviews. BMJ.

[21] Whiting PF, Rutjes AW, Westwood ME, Mallett S, Deeks JJ, Reitsma JB (2011). QUADAS-2: a revised tool for the quality assessment of diagnostic accuracy studies. Ann Intern Med.

[22] Björkman AS, Gauffin H, Persson A, Koskinen SK (2022). Sensitivity of DECT in ACL tears. A prospective study with arthroscopy as reference method. Acta Radiologica Open.

[23] Bai R, Huang JC, He XH, Huang WJ, Guo YX, Li PY (2022). Clinical comparative analysis of low-dose dual-energy CT in the diagnosis of anterior cruciate ligament injury of knee joint. Chinese Journal of CT and MRI.

[24] Ren R, Song Q, Zhang ZL, Chen L, Zhai CB (2020). Application of dualsource CT imaging in diagnosis of anterior cruciate ligament. Injury J Binzhou Med Univ.

[25] Cj BAO (2018). Clinical evaluation and analysis of dual-source CT for imaging cruciate ligament injury of knee joint. Chinese Manipulation & Rehabilitation Medicine.

[26] Suo YM, Song XL, Qiu XH, Zhang HQ (2016). Comparative research on the diagnosis of cruciate Hgament injury by dual·energy CT and MRI. Anhui Med J.

[27] Pang YM, Mou XM (2015). Clinical evaluation of dual-energy CT in patients with anterior cruciate ligament injury of knee joint. Medical Journal of Chinese Peopleˊs Health.

[28] Cao JX, Kong XQ, Wang YM, Yang C, Zhuang FG, Zhang Y (2015). Feasibility of dual-energy CT in the diagnosis of anterior cruciate ligament injury of knee joint. Computerized Tomography Theory and Applications.

[29] Bai R, Qiao GQ, Guo YX, He XH, Luo DS, Peng GM (2014). Diagnosis of acute injury for anterior cruciate ligament by dua-energy computed tomograph. Mil Med JS Chin.

[30] Rajiah P, Sundaram M, Subhas N (2019). Dual-Energy CT in Musculoskeletal Imaging: What Is the Role Beyond Gout?. AJR Am J Roentgenol.

[31] Mallinson PI, Coupal TM, McLaughlin PD, Nicolaou S, Munk PL, Ouellette HA (2016). Dual-energy CT for the musculoskeletal system. Radiology.

[32] Cheraya G, Sharma S, Chhabra A (2022). Dual energy CT in musculoskeletal applications beyond crystal imaging: bone marrow maps and metal artifact reduction. Skeletal Radiol.

[33] Sun C, Miao F, Wang XM, Wang T, Ma R, Wang DP (2008). An initial qualitative study of dual-energy CT in the knee ligaments. Surg Radiol Anat.

[34] Gruenewald LD, Booz C, Martin SS, Mahmoudi S, Yel I, Eichler K (2024). Diagnostic performance of modern computed tomography in cruciate ligament injury detection: a comprehensive study. Eur J Radiol.

[35] Glazebrook KN, Brewerton LJ, Leng S, Carter RE, Rhee PC, Murthy NS (2014). Case-control study to estimate the performance of dual-energy computed tomography for anterior cruciate ligament tears in patients with history of knee trauma. Skeletal Radiol.

[36] Fickert S, Niks M, Dinter DJ, Hammer M, Weckbach S, Schoenberg SO (2013). Assessment of the diagnostic value of dual-energy CT and MRI in the detection of iatrogenically induced injuries of anterior cruciate ligament in a porcine model. Skeletal Radiol.

[37] Parikh R, Mathai A, Parikh S, Chandra Sekhar G, Thomas R (2008). Understanding and using sensitivity, specificity and predictive values. Indian J Ophthalmol.

